# The roles of cell wall inhibition responsive protein CwrA in the pathogenicity of *Staphylococcus aureus*

**DOI:** 10.1080/21505594.2024.2411540

**Published:** 2024-10-02

**Authors:** Weihua Han, Yanghua Xiao, Li Shen, Xinru Yuan, Jingyi Yu, Haojin Gao, Rongrong Hu, Junhong Shi, Bingjie Wang, Jiao Zhang, Peiyao Zhou, Cailing Wan, Yu Huang, JianBo Lv, Fangyou Yu

**Affiliations:** a Department of Clinical Laboratory, Shanghai Pulmonary Hospital, School of Medicine, Tongji University, Shanghai, People’s Republic of China; b Shanghai Institute of Immunity and Infection, Chinese Academy of Science, Shanghai, People’s Republic of China

**Keywords:** *Staphylococcus aureus*, cell wall inhibition responsive protein, biofilm, hemolysin, VraSR, SaeRS

## Abstract

The ability to form robust biofilms and secrete a diverse array of virulence factors are key pathogenic determinants of *Staphylococcus aureus*, causing a wide range of infectious diseases. Here, we characterized *cwrA* as a VraR-regulated gene encoding a cell wall inhibition-responsive protein (CwrA) using electrophoretic mobility shift assays. We constructed *cwrA* deletion mutants in the genetic background of methicillin-resistant *S. aureus* (MRSA) and methicillin-sensitive *S. aureus* (MSSA) strains. Phenotypic analyses indicated that deletion of *cwrA* led to impaired biofilm formation, which was correlated with polysaccharide intercellular adhesin (PIA). Besides, the results of real-time quantitative PCR (RT-qPCR) and β-galactosidase activity assay revealed that CwrA promoted biofilm formation by influence the *ica* operon activity in *S. aureus*. Furthermore, *cwrA* deletion mutants released less extracellular DNA (eDNA) in the biofilm because of their reduced autolytic activity compared to the wild-type (WT) strains. We also found that *cwrA* deletion mutant more virulence than the parental strain because of its enhanced hemolytic activity. Mechanistically, this phenotypic alteration is related to activation of the SaeRS two-component system, which positively regulates the transcriptional levels of genes encoding membrane-damaging toxins. Overall, our results suggest that CwrA plays an important role in modulating biofilm formation and hemolytic activity in *S. aureus*.

## Introduction

*Staphylococcus aureus*, one of the most commonly isolated human pathogens, is responsible for a wide spectrum of diseases, ranging from benign skin infections to fatal disorders such as bacteremia, endocarditis, osteomyelitis, and toxic shock syndrome [1]. Biofilm development and virulence factor secretion play vital roles in the pathogenicity of *S. aureus*. After colonization on both biotic and abiotic surfaces, *S. aureus* could form a three-dimensional complex community of bacteria within a layer of exopolysaccharide (EPS) termed as “biofilm,” which was often associated with the infection of indwelling medical devices [2]. In addition, *S. aureus* produces a diverse array of virulence factors such as hemolysins, microbial surface components recognizing adhesive matrix molecules (MSCRAMMs), leukotoxins, Protein A exfoliative toxins, staphylococcal enterotoxins, and toxic-shock syndrome toxin-1, which allow *S. aureus* to survive under host immune responses and cause a wide range of severe clinical infections [3].

*S. aureus* has a typical Gram-positive cell wall, which is composed of peptidoglycan (PG), the anionic polymers lipoteichoic acid (LTA) and wall teichoic acid (WTA) [4]. Complex molecular networks related to cell wall stress responses regulate *S. aureus* pathogenicity [5]. Cell wall stress stimulation (CWSS) is a cluster of genes induced when *S. aureus* is exposed to a broad range of cell wall synthesis inhibitors such as β-lactams, glycopeptides, bacitracin, and D-cycloserine [6]. One of these genes, the hypothetical protein-encoding gene annotated as *cwrA*, SA2343, has been reported to be consistently upregulated in vancomycin-adapted strains [7]. Additionally, it has been found to be significantly overexpressed in a vancomycin-intermediate *S. aureus* (VISA) isolate compared to its parent-sensitive strain in response to vancomycin treatment [8]. Acquisition of vancomycin resistance in *S. aureus* is often accompanied by a decrease in virulence, such as reduced coagulase activity, decreased pigment formation, and less or no hemolysis [9–11]. However, it remains to be confirmed whether the induced expression of *cwrA* directly contribute to the alteration of the virulence phenotype in VISA.

Previous transcriptomic studies have revealed that *cwrA* may be positively regulated by VraSR (a vancomycin-resistance-associated sensor/regulator), a two-component system consisting of a histidine kinase sensor VraS and response regulator VraR that can activate the expression of CWSS [12]. VraSR is involved in the virulence of *S. aureus* through cross-regulation with the accessory gene regulator (*agr*) quorum sensing system [13,14], which is responsible for the expression of various virulence factors (alpha-hemolysin, leukocidins, γ-hemolysin, and phenol-soluble modulins) [15]. Furthermore, VraSR modulates biofilm formation by *Staphylococcus epidermidis* in an *ica*-dependent manner [16]. The *ica* (intercellular adhesion) locus is necessary for the biosynthesis of fully functional polysaccharide intercellular adhesin (PIA), which is a major component of EPS [17]. Therefore, we hypothesized that the cell wall inhibition-responsive protein (CwrA) might participate in VraSR-mediated virulence factor production and biofilm formation.

In this study, we demonstrated that VraR could directly bind to the promoter region of *cwrA*. Subsequently, we examined the function of CwrA by comparing the traits of the wild-type strains (WT) with their respective *cwrA* deletion mutants (Δ*cwrA*). Our results showed that Δ*cwrA* mutants had impaired biofilm formation in vitro and reduced adherence to both A549 cells and murine nasal tissue. Additionally, we verified that CwrA enhanced *S. aureus* biofilm formation by promoting PIA biosynthesis and extracellular DNA (eDNA) release within the biofilm. Furthermore, CwrA inhibited *S. aureus* hemolytic activity by suppressing the SaeRS system. Thus, CwrA seems as a mediator involved in the crosstalk between VraSR and SaeRS two-component system (TCSs) and contributes to attenuating the virulence of *S. aureus*. This is the first study to characterize the crucial role of CwrA in *S. aureus* biofilm formation and virulence factor expression. These findings improve our understanding of the global regulatory networks of *S. aureus*.

## Materials and methods

### Ethics statement

This study was approved by the Ethics Committee of the Shanghai Pulmonary Hospital, School of Medicine, Tongji University, Shanghai, China. All animal experiments were carried out in strict accordance with the ARRIVE guidelines (https://arriveguidelines.org/). In addition, the guidelines approved by the Ethics Committee of the Shanghai Pulmonary Hospital of Tongji University School, Tongji University, Shanghai (Project number: K20-151Y) were strictly followed for all animal experiments.

### Bacterial strains, plasmids and culture conditions

The bacterial strains and plasmids used in the present study are listed in Table S1. Strains N315, USA300 (LAC), SA113, and Newman were used in this study. N315 and USA300 (LAC) belong to methicillin-resistant *S. aureus* (MRSA), whereas SA113 and Newman belong to the methicillin-sensitive *S. aureus* (MSSA) strains. SA113 was a gift from the Department of Infectious Diseases and Key Laboratory of Endogenous Infection, Shenzhen Nanshan People’s Hospital (Shenzhen, China). Unless otherwise noted, *S. aureus* strains were cultured in tryptic soy broth (TSB, Oxoid) medium at 37 °C with shaking at 220 rpm or on tryptic soy agar (TSA, Oxoid) at 37 °C. *Escherichia coli* (*E. coli*) strains were cultured in Luria-Bertani (LB, Oxoid) medium at 37 °C with shaking at 220 rpm, or on LB agar plates at 37 °C. Appropriate antibiotics were used at the following concentrations for antibiotic selection and plasmid maintenance: for *S. aureus*, chloramphenicol (Solarbio) at 10 μg/ml, erythromycin (MedChemExpress) at 10 μg/ml, and anhydrotetracycline (Sigma-Aldrich) at 2 μg/ml; for *E. coli*, ampicillin (Sangon Biotech) at 100 μg/ml, and kanamycin (Sangon Biotech) at 50 μg/ml.

### Construction of *S. aureus* gene deletion mutants

To construct the *cwrA* null mutant, allelic replacement was performed as previously described [18]. The upstream fragment (810bp) and downstream fragment (805bp) were amplified from *S. aureus* N315 genomic DNA using primers *cwrA*-up-F/*cwrA*-up-R and *cwrA*-down-F/*cwrA*-down-R (Table S2), respectively, and then these two fragments were ligated by overlapping PCR. The resultant ligated product was used for recombination with the plasmid pKOR1 [19]. The plasmid pKOR1-*cwrA* was successively introduced into *E. coli* DH5a and DC10B, and sequencing was performed to ensure that no unwanted mutations occurred. The resulting plasmid was then transferred by electroporation to *S. aureus* N315, SA113, and Newman. Allelic replacement was performed to induce replacement of the native gene with the spliced allele. Deletion of the target gene was confirmed by PCR, first-generation sanger DNA sequencing, and real-time fluorescence quantitative PCR (RT-qPCR) (Supplementary Figure 1). We used a similar strategy to construct Δ*saeR* mutants in *S. aureus* SA113 genetic background. The primers used for constructing the Δ*saeR* mutants are shown in Table S2. To construct the SA113-Δ*cwrA*-Δ*saeR* mutant, pKOR1-*saeR* was transferred to the SA113-Δ*cwrA* strain by electroporation.

### Construction of *S. aureus* gene complementary mutants

To construct plasmid pLi50-*cwrA*-C, a 421 bp DNA fragment containing *cwrA* and its predicted promoter was amplified from *S. aureus* N315 genomic DNA using the primers *cwrA*-C-F and *cwrA*-C-R (Table S2). The *cwrA* DNA fragment and plasmid pLi50 were digested with *BamHI* and *HindIII*, and then ligated with T4 ligase, yielding plasmid pLi50-*cwrA*-C. The plasmid pLi50-*cwrA*-C was then successively introduced into *E. coli* DH5a and DC10B and a sequencing analysis was performed to ensure that no unwanted mutations was presented. The resulting plasmid was then transferred by electroporation into *S. aureus* N315-Δ*cwrA*, SA113-Δ*cwrA*, and Newman-Δ*cwrA*. Complementation of target gene was confirmed by RT-qPCR (Supplementary Figure 1).

### Construction of icaR-lacZ transcriptional fusion

Constructing *icaR-lacZ* transcriptional fusion was performed as previously described [20]. The promoter region of *icaR* (−217 to 26 of the start codon) was amplified from *S. aureus* N315 genomic DNA using the primers *icaR*-pro-F and *icaR*-pro-R (Table S2). Subsequently, the promoter fragment and plasmid pCL-*lacZ* were digested with *EcoRI* and *KnpI*. Digested DNA fragments were ligated into the pCL-*lacZ* plasmid using T4 ligase, and the resulting plasmids were introduced into *E. coli* DH5a. After confirmation by DNA sequencing, these constructs were electroporated into *S. aureus* RN4220 and subsequently into *S. aureus* N315, SA113, and their derivatives using the bacteriophage Ø85.

### Minimum inhibitory concentration (MIC) assays

Overnight cultures of *S. aureus* were diluted 1:200 in 4 ml fresh TSB medium and cultured at 37 °C with shaking (220 rpm). The exponential phase bacterial suspension was diluted with Mueller-Hinton II broth (cation-adjusted, BD 212,322) to 1.5 × 10^6^ CFU/ml. Each 100 μl bacterial dilution was distributed in 96-well plates, and the antibiotics were serially diluted two-fold. After incubating at 37 °C for 18–24 h, MIC values were determined, which were defined as the lowest compound concentration to inhibit bacterial growth completely. *S. aureus* ATCC29213 was used as a quality control, and interpretative breakpoints were determined according to CLSI2021-M100-ED31.

### Total RNA isolation and cDNA synthesis

Bacteria were grown to mid-exponential phase at 37 °C in TSB medium. After centrifugation at 12,000 × g for 2 min, cells were resuspended in TE buffer containing 20 mg/ml lysostaphin and incubated at 37 °C for 20 min. After digestion, total RNA was extracted according to the manufacturer’s instructions (Spin Column Bacteria Total RNA Purification Kit; Sangon Biotech). RNA was reverse-transcribed into cDNA using a cDNA synthesis kit with gDNA Eraser (Takara).

### Real-time fluorescence quantitative PCR analysis

Real-time quantitative PCR was performed using TB Green^TM^ Premix Ex Taq^TM^ II (Takara) on a QuantStudio^TM^ 5 Real-Time PCR System (Applied Biosystems). The primers used for RT-qPCR are listed in Table S2. Relative expression values were calculated using the 2^−∆∆CT^ method, with the internal control gene *gyrB* as the normalizer. Three biological and three technical replicates were performed for each gene.

### β-galactosidase activity assay

β-galactosidase activity assay was performed as previously described [14]. Overnight cultures of *S. aureus* were diluted 1:200 in 4 ml fresh TSB medium containing chloromycetin and cultured at 37 °C with shaking (220 rpm) for 8 h. After the optical density at 600 nm (OD_600_) was measured, cells were harvested by centrifugation at 12,000 × *g* for 2 min and resuspended in 100 μl ABT-LSA buffer (60 mm K_2_HPO_4_, 40 mm KH_2_PO_4_, 100 mm NaCl, 0.1 % (v/v) Triton X-100, and 50 μg/ml lysostaphin). After lysis at 37 °C for 30 min, 100 μl ABT buffer (60 mm K_2_HPO_4_, 40 mm KH_2_PO_4_, 100 mm NaCl) and 100 μl o-nitrophenyl-β-D-galactopyranoside (5 mg/ml) were added into reactions. Following that, the mixtures were kept at 37 °C until the solution changed to yellow. Subsequently, 1 ml of 1 M Na_2_CO_3_ was introduced to halt the reaction, and the reaction time (T) was documented. The absorbance of the supernatant was measured at 420 nm (OD_420_) after centrifugation at 12,000 × *g* for 2 min. β-Galactosidase units were calculated using the following formula:units = (1000 × OD_420_)/(T × V ×OD_600_).

### Cloning, expression and purification of His_6_-VraR

His_6_-VraR protein was cloned, expressed and purified as previously described [20]. Firstly, a 641 bp DNA fragment containing *vraR* was amplified from *S. aureus* N315 genomic DNA using the primers His-*vraR*-F and His-*vraR*-R (Table S2). The plasmid pET28a and the *vraR* DNA fragment were digested with *BamHI* and *XhoI*, and the post-digested fragment and plasmid were ligated with T4 ligase (Supplementary Figure 2A). The resultant pET28a-His_6_-*vraR* was introduced into *E. coli* DH5a and sequencing analysis was performed to ensure that no unwanted mutations occurred. Finally, the resulting plasmid was transformed into *E. coli* BL21 (DE3) cells for His_6_-VraR protein expression and purification.

Overnight LB cultures of *E. coli* BL21 (DE3) carrying the plasmid pET28a-His_6_-*vraR* were diluted 1:100 in 5 L fresh LB medium supplemented with 50 μg/ml kanamycin and grown at 37 °C with shaking at 220 rpm to an OD_600_ value of 0.6. 0.5 mm β-D-1-thiogalactopyranoside (IPTG, Sangon) was added for 16 h at 16°C to induce protein expression. Then, cells were harvested by centrifugation at 4 °C and resuspended in 40 ml lysis buffer (20 mm Tris-HCl (pH 8.0), 1 mm dithiothreitol (DTT) and 0.5 M NaCl). The resuspended cells were lysed using a high-pressure cell crusher and centrifuged at 13,000 × g for 1 h at 4 °C to remove insoluble material and the membrane fraction. Supernatants were loaded onto a HisTrap HP column (GE Healthcare Life Sciences). Non-specifically bound hetero-proteins were eluted by using buffers containing 10 mm, 20 mm and 40 mm imidazole, and His_6_-VraR was eluted using a buffer containing 300 mm imidazole (50 mm Tris-HCl (pH 8.0), 1 mm DTT, 300 mm NaCl and 300 mm imidazole). To remove the imidazole, the collected fractions were loaded onto the HiTrap Desalting 5 × 5 ml (Sephadex G-25 S) (GE Healthcare Life Sciences), and the purified His_6_-VraR protein was verified by SDS-PAGE followed by Coomassie blue staining (Supplementary Figure 2B). Finally, the protein concentration of His_6_-VraR was determined using a NanoDrop 2000 (Thermo Fisher Scientific) by A_280 nm_.

### Electrophoretic mobility shift assay (EMSA)

A 359 bp DNA fragment covering the promoter region of *cwrA* was amplified from *S. aureus* N315 genomic DNA using primers *cwrA*-EMSA-F and *cwrA*-EMSA-R (Table S2). The DNA fragment was purified using a purification kit (Omega Bio-tek) and labeled with biotin. To verify the interaction between VraR and the promoter regions of *cwrA*, electrophoretic mobility shift assay (EMSA) was carried out. In this experiment, reactions in 20 μl volume containing 2 ng/μl *cwrA* promoter DNA fragments were mixed with various concentrations of His_6_-VraR in binding buffer (10 mm Tris-HCl (pH 7.5), 50 mm KCl, 1 mm DTT, 5 mm MgCl2, 0.05 % Nonidet® *p*-40, 2.5 % glycerol, and 50 mm acetyl phosphate). Followed that reactions were incubated at 25 °C for 30 min, the mixtures were separated by electrophoresis on a 6 % non-denaturing polyacrylamide gel and blotted onto a positively charged nylon membrane (Millipore, Bedford, MA, USA). The blots were incubated with streptavidin-horseradish peroxidase antibody (Beyotime) and visualized using enhanced chemiluminescence (ECL, Beyotime).

### Growth curve

Overnight cultures of *S. aureus* were adjusted to a turbidity equivalent to that of the McFarland standard 0.5, diluted 1:200 in fresh TSB medium, and incubated at 37 °C with shaking (220 rpm) for 24 h. The optical density at 600 nm (OD_600_) was measured every 1 h for 24 h using a microplate reader (OY Growth Curves). All experiments were performed in triplicate.

### Biofilm semi-quantitative assay

Overnight cultures of *S. aureus* were diluted 1:100 in fresh TSB medium with 0.5 % glucose and incubated in 96-well polystyrene microtiter plates at 37 °C for 24 h. Afterwards, supernatants were discarded and the wells were gently washed three times with phosphate-buffered saline (PBS, Sangon, Shanghai). The bacterial cells were fixed with methanol for 10 min and stained with 1 % crystal violet for another 10 min. Subsequently, 30% acetic acid was used to dissolve the biofilms, and OD_600_ was measured using a microplate reader (Hiwell-Diatek Instruments). All samples were tested in triplicate and repeated three times.

### Confocal laser scanning microscopy (CLSM)

Overnight cultures of *S. aureus* were diluted 1:100 in fresh TSB medium with 0.5 % glucose and incubated in glass bottom cell culture dishes (Biosharp). After 24 h of static incubation at 37 °C to form biofilms, the supernatants were discarded, and the dishes were washed with PBS for three times. Then, the 500 μl PBS containing 0.02 % SYTO9 (Thermo Fisher) and 0.067 % propidium iodide (Thermo Fisher) was added to each dish to stain biofilms in the dark. Then, using a 63 × 1.4 numerical aperture oil immersion lens objective, the samples were scanned using CLSM (TCS SP5; Leica).

### Cell adhesion assay

The A549 lung epithelial cells were grown in DMEM medium supplemented with 10 % fetal bovine serum at 37 °C and 5 % CO_2_. Wells containing 5 × 10^5^ cells were washed twice with sterile phosphate-buffered saline (PBS). After that, *S. aureus* strains incubated for 6 h at 37 °C to the logarithmic growth phase were adjusted to a turbidity equivalent to that of a 0.5 McFarland standard and resuspended in DMEM medium without serum. The prepared A549 cells were co-incubated with bacteria at a multiplicity of infection (MIO) of 10:1 for 2 h. Subsequently, the supernatant was discarded, and the wells were washed three times with sterile PBS to remove unattached bacteria. Then the A549 cells were dissociated with 200 μl trypsin-EDTA for 3 min and lysed with 0.05 % Triton X-100 for 5 min. Finally, we determined the bacterial CFU by plating serial dilutions of epithelial cell lysates on TSA plates.

### Mouse nasal mucosa colonization assay

The mouse nasal mucosa colonization assay was performed as previously described with slight modifications [21]. Briefly, 6–8 weeks old female BALB/c mice were randomly divided into three groups of six mice each. Overnight cultures of *S. aureus* were diluted 1:200 in fresh TSB medium and incubated at 37 °C with shaking (220 rpm) for 6 h to the logarithmic growth phase. After harvested by centrifugation at 4,000 × *g* for 5 min, the bacteria were washed twice and adjusted to a turbidity equivalent to that of a 0.5 McFarland standard with PBS. Then, 50 μl PBS containing 1.5 × 10^9^ bacterial cells were slowly dropped into the nasal cavities of the mice. The mice were euthanized 24 h later and their nasal tissues were excised and homogenized. The homogenates were diluted and dropped onto TSA plates to determine CFU counts.

### Enzyme-linked dot immunoblot assay for PIA

PIA in the biofilm of *S. aureus* was semi-quantified by dot blot assay with wheat germ agglutinin (WGA-horseradish peroxidase [HRP] conjugate), as described previously [22]. Briefly, overnight cultures of *S. aureus* were diluted 1:100 in 3 ml fresh TSB medium with 0.5 % glucose and incubated in 6-well polystyrene microtiter plates at 37 °C for 24 h. After supernatants were discarded, biofilms collected from the bottom of the wells were resuspended in 500 μl 0.5 M EDTA (Sangon) and centrifuged (12,000 × *g*, 2 min) after heating at 100 °C for 10 min. Then, the supernatants containing PIA were treated with proteinase K (20 mg/ml) at 37 °C for 2 h. Afterwards, PIA extracts were spotted onto the nitrocellulose filter membranes (Millipore). After being dry, the membranes were blocked with 5 % (w/v) skim milk at 4 °C overnight and subsequently incubated with WGA-HRP at room temperature for 2 h. HRP activity was visualized using enhanced chemiluminescence (ECL, Beyotime).

### Purification and quantification of eDNA

Purification and quantification of eDNA were performed as previously described [22]. In brief, overnight cultures of *S. aureus* were diluted 1:100 in 2 ml fresh TSB medium with 0.5 % glucose and incubated in 6-well polystyrene microtiter plates at 37 °C for 24 h. Supernatants containing extracellular material were collected by centrifugation at 4,000 × *g* for 5 min and filtered using a 0.22-μm-pore-size polyether sulfone membrane to remove the bacterial cells. Each supernatant (500 μl) was mixed with 500 μl 0.5 M EDTA (Sangon), and then the eDNA was extracted with 700 μl of phenol/chloroform/isoamyl alcohol (25:24:1) and 700 μl chloroform/isoamyl alcohol (24:1). Subsequently, the 900 μl aqueous phase of each sample was mixed with 100 μl 3 M sodium acetate and stored at 4 °C overnight. The eDNA precipitate was washed twice with 75 % (v/v) ethanol, air-dried, and dissolved in 500 μl nuclease-free water. A Nanodrop 2000 spectrophotometer (Thermo Fisher Scientific) was used to quantify eDNA. The relative OD_600_ of each biofilm was determined by normalizing the eDNA concentration.

### Triton X-100-induced autolysis assay

Overnight cultures of *S. aureus* were diluted 1:200 into TSB supplement with 1 M NaCl and incubated at 37 °C with shaking at 220rpm until mid-exponential phase (OD_600_ = 0.6-0.8). The cells were harvested by centrifugation (4,000 × *g*) at 4 °C for 20 min. After being washed twice with cold sterile deionized water, the cells were resuspended to OD_600_ = 1.0 in 0.05 M Tris-HCl (pH7.2) containing 0.05% (v/v) Triton X-100 followed by incubation at 37 °C with shaking (220rpm) for three hours, and the progressive decrease in absorbance (OD_600_) was monitored every 30 min using a microplate reader (Hiwell-Diatek Instruments). Each data point represents the mean ± standard deviation of three independent experiments.

### Transmission electron microscopy (TEM)

Transmission electron microscopy (TEM) was performed as previously described with slight modifications [23]. Overnight cultures of *S. aureus* were collected by centrifugation at 4,000 × *g* for 5 min to avoid cell damage. The cells were immediately resuspended in 0.1 M phosphate buffer (pH 7.4) containing 2.5 % (v/v) glutaraldehyde overnight at 4 °C for fixation. Samples were collected by centrifugation at 4,000 × *g* for 5 min, and agar embedding was performed to easily process the following operation. After being washed with 0.1 M phosphate buffer (pH 7.4) for three time at 4 °C, they were fixed again with 1 % (v/v) OsO4 for 1 h at 4 °C. Afterwards, specimens were washed with 0.1 M phosphate buffer (pH 7.4) for three times and stained with 3 % (v/v) uranyl acetate for 3 h at 4 °C. Before being infiltrated and embedded in acetone and resin, the samples underwent dehydration in a series of graded ethanol solutions. After polymerization at 60 °C for 48 h, thin slices were cut with a diamond knife using an LKB ultramicrotome (LKB Instruments). The slices were positioned on uncoated 200-mesh copper grids prior to staining with lead citrate. Sections were subsequently observed under a Tecnai F20 G2 (FEI Company) Transmission Electron Microscope operated at 100 kV.

### Mouse skin abscess model

6-8 weeks old female BALB/c mice were housed in a specific pathogen-free facility and the libitum diet was given to them. Overnight cultures of *S. aureus* were diluted 1:200 in fresh TSB medium and incubated at 37 °C with shaking (220 rpm) for 6 h to the logarithmic growth phase. After harvested by centrifugation at 4,000 × *g* for 5 min, the bacteria were washed twice and adjusted to a turbidity equivalent to that of a 0.5 McFarland standard with PBS. Then, mice were injected subcutaneously in the shaved flank with 100 μl PBS containing 1.5 × 10^9^ bacterial cells suspended. After 24 h, abscess sizes were measured with calipers using the formula: A= *Π* × (*L* × *W*). The bacterial load in the abscess was determined by serial dilutions and spotted on TSA plates. Skin abscess tissues were dissected into pathological sections 24 h post-infection.

### Hemolytic activity assay

Overnight cultures of *S. aureus* were diluted 1:200 in 4 ml fresh TSB medium and incubated at 37 °C with shaking (220 rpm) for 16 h. After being adjusted to the turbidity equivalent to that of a 0.5 McFarland standard with sterile PBS, 10 μl bacterial suspensions were spotted onto 5 % (v/v) sheep blood agar plates (Kemajia Microbe Technology). Zones of clearance surrounding bacterial colonies indicate hemolytic activity. To quantitatively compare hemolytic activity of perspective strains, 100 μl bacterial supernatant collected by centrifugation at 4,000 × *g* for 5 min was mixed with 900 μl PBS containing 3 % (v/v) healthy adult blood cells, and the mixtures were incubated at 37 °C with shaking (220 rpm) for 1 h. Afterwards, the supernatants were collected by centrifugation at 4,000 × *g* for 5 min, and the absorbance at 600 nm was measured with a microplate reader (Hiwell-Diatek Instruments). PBS and 0.5 % (v/v) Triton X-100 were used as negative and positive controls, respectively, and the hemolysis ratio was calculated using the following formula: hemolysis (%) = (A_sample_-A_PBS_)/(A_TritonX-100_-A_PBS_). Three independent experiments were performed.

### Western blot analysis

Western blotting analysis of Hla was performed as previously described [24]. Overnight cultures of *S. aureus* were diluted 1:200 in 4 ml fresh TSB medium and incubated at 37 °C with shaking (220 rpm) for 24 h. Then, bacterial culture supernatants were collected by centrifugation at 4,000 × *g* for 5 min, and Bradford assays were used to determine the protein concentration of each sample to maintain the total amount of protein loaded for SDS-PAGE was consistent. After mixing with Omni-EasyTM Protein Sample Loading Buffer (EpiZyme Biotechnology Co., Ltd., Shanghai, China), samples were heated at 95 °C for 10 min and separated by 12.5 % (w/v) SDS-PAGE. Subsequently, the proteins on the gel were transferred onto PVDF (Bio-Rad) membranes. The membranes were blocked with 5 % (w/v) skim milk at 4 °C overnight and subsequently incubated with a rabbit anti-Hla IgG antibody (Sigma, 1:2500 dilution) at 4 °C overnight. Following incubation with Goat Anti-Rabbit IgG HRP (Biosharp, 1:2500 dilution) at room temperature for 2 h, the resulting chemiluminescence was detected using a Tanon-5200 multi (Tanon Science & Technology Co., Ltd.).

### RNA-Seq and data analysis

Total RNA was isolated as previously described using a Qiagen RNeasy kit, following the manufacturer’s instructions. mRNA was purified from total RNA using probes to remove rRNA and then used to generate a cDNA library. Library quality was assessed using an Agilent Bioanalyzer 2100 system. Clustering of the index-coded samples was performed on a cBot Cluster Generation System using the TruSeq PE Cluster Kit v3-cBot-HS (Illumina) according to the manufacturer’s instructions. After cluster generation, the library preparations were sequenced on an Illumina NovaSeq platform and 150 bp paired-end reads were generated.

Bacterial RNA-seq reads were mapped to the *S. aureus* NCTC8325 genome (GenBank: CP000253) using Bowtie 2 (version 2.2.3) [25] with two mismatches allowed. Prior to differential gene expression analysis, for each sequenced library, the read counts were adjusted using the edgeR program package through one scaling normalized factor. Differential expression analysis of the two groups was performed using the DESeq R package (version 1.18.0). An adjusted P-value ≤0.05 and log2 (fold change) ≥1 was set as the threshold for significantly differential expression. To show the overlapping cluster that was significantly changed in both *cwrA* and *saeR* mutants, the threshold for significantly differential expression was set as adjusted P-value ≤0.05, and log2 (fold change) ≥2. We used the KOBAS software to test the statistical enrichment of expression genes in the KEGG pathways. Gene Ontology (GO) enrichment analysis of differentially expressed genes was performed using the Goseq R package, in which the gene length bias was corrected. GO terms with corrected *p* values less than 0.05 were considered significantly enriched by differentially expressed genes.

### Statistical analysis

Statistical analysis was conducted using the GraphPad Prism version 8.00 software. Two-tailed Student’s *t* test was used to analyze the statistical difference between two groups, and one-way or two-way analysis of variance (ANOVA) was used to analyze the statistical difference between multiple groups after passing the normal distribution test. The Shapiro-Wilk test was used to verify data normality. Error bars in the figures represent the standard deviation of the dataset (mean ± standard deviation). *p < 0.05, **p < 0.01, ***p < 0.001, ****p < 0.0001.

## Results

### VraR protein can directly bind to the promoter region of cwrA

**Figure 1.**
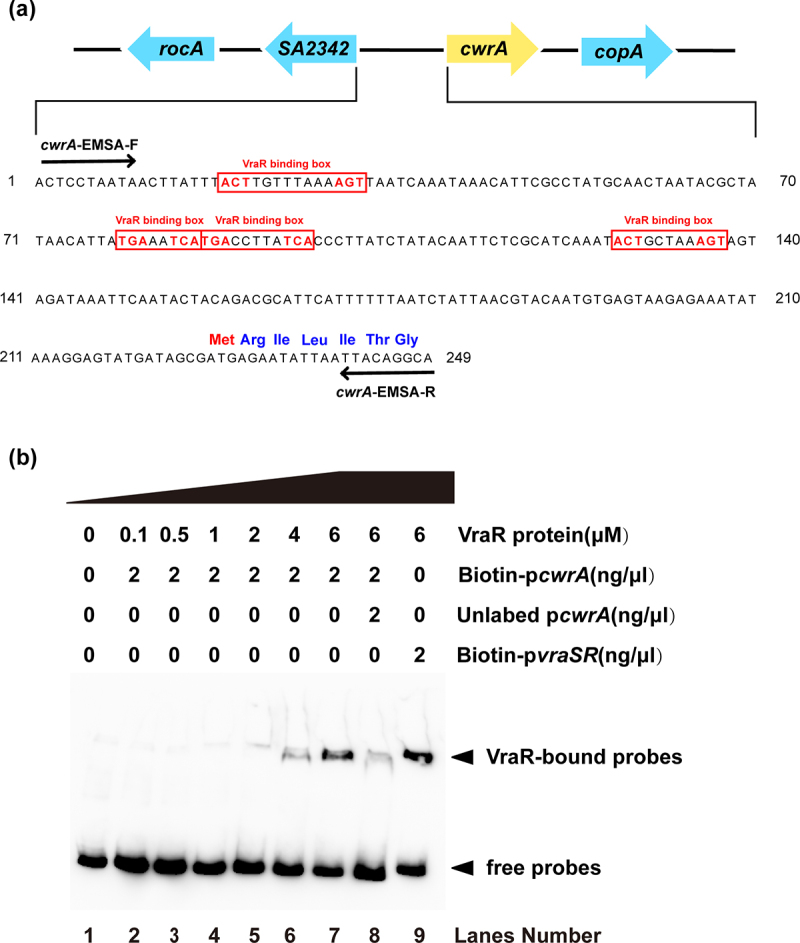
VraR could regulate the transcription of *cwrA*. (a) The VraR binding boxes were found in the putative region of *cwrA* and are highlighted in red frames. (b) Dose-dependent band shifts caused through specific VraR binding to the putative promoter region of *cwrA*.

Bioinformatic analysis showed that the VraR-binding boxes (ACT[X]_n_AGT or TGA[X]_n_TCA) were present in the upstream region of *cwrA* gene in the genome of *S. aureus* N315 (Figure 1a). To further investigate the mechanism of regulation of *cwrA* by VraR, His-tagged VraR protein was expressed and purified to carry out an EMSA with DNA probes containing the putative promoter sequences of *cwrA*. The 249-bp DNA fragment upstream of *cwrA* that labeled biotin (Biotin-p*cwrA*) formed a shifted complex with phosphorylated VraR in a dose-dependent manner (Figure 1b lanes 2 to 7). As a specific competitor, the addition of the same amount of unlabeled p*cwrA* affected the formation of the shifted complex formation (Figure 1b lane 8). The 298-bp DNA fragment of *vraSR* promoter labeled with biotin was incorporated into this assay as a positive control (Figure 1b lane 9). These results suggested that VraR could directly bind to the promoter region of *cwrA* and may serve as an upstream regulator influencing the transcriptional level of *cwrA*.

### The *S. aureus* cwrA gene affects in Vitro biofilm formation

**Figure 2.**
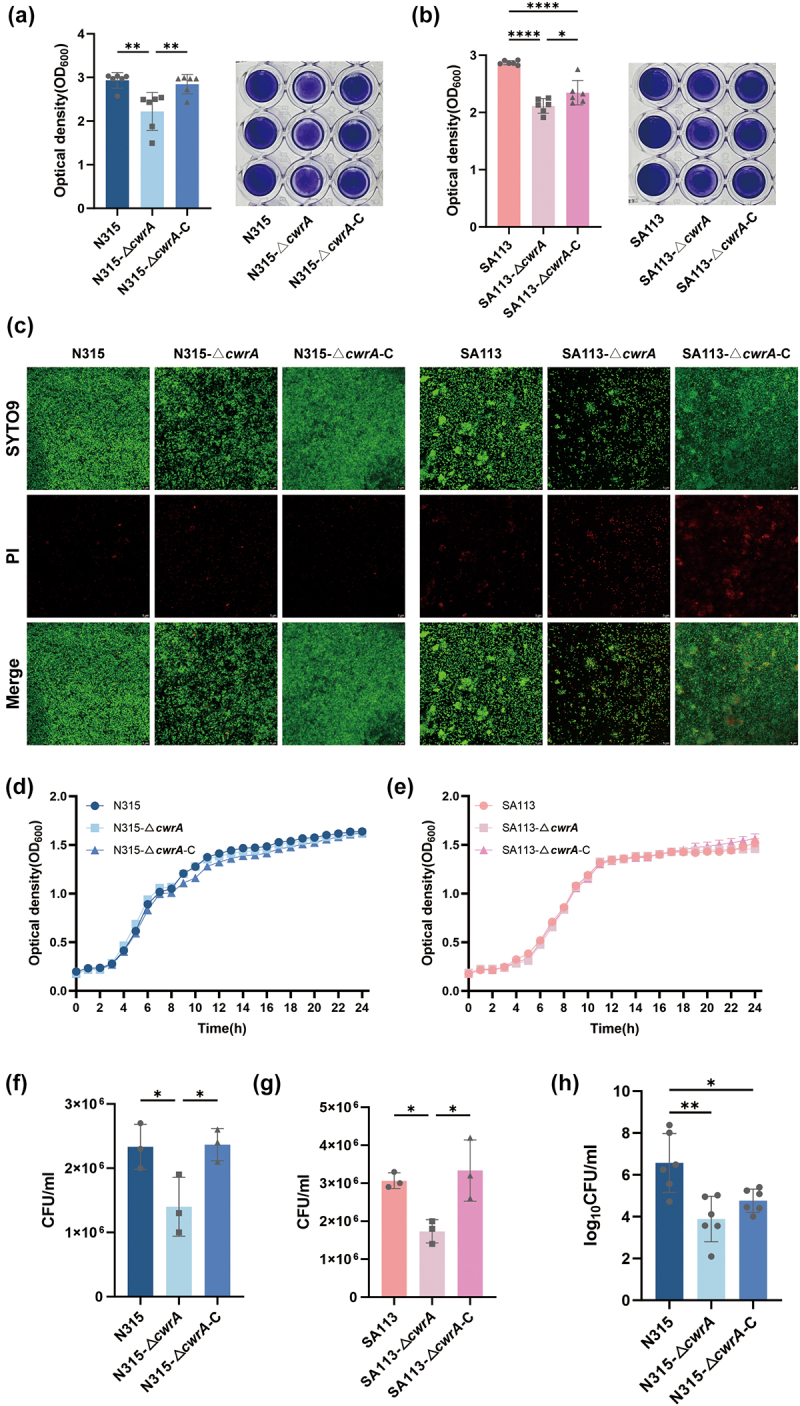
In vitro biofilm-forming ability and in vivo adhesion force of *S. aureus* N315, SA113 and their isogenic mutant strains. (a) The biofilm formation ability of *S. aureus* N315 and its isogenic mutant strains on polypropylene 96-well plates (right), and the optical density values at 600 nm were measured after the biofilms were dissolved with acetic acid (left). (b) The biofilm formation ability of *S. aureus* SA113 and its isogenic mutant strains on polypropylene 96-well plates (right), and the optical density values at 600 nm were measured after the biofilms were dissolved with acetic acid (left). (c) The 24-h biofilms of tested strains were visualized by using live (SYTO9)/dead (PI) viability staining under a CLSM (scale bars, 5 μm). (d) Growth curves of *S. aureus* N315 and its isogenic mutant strains. (e) Growth curves of *S. aureus* SA113 and its isogenic mutant strains. (f) The adhesion force of *S. aureus* N315 and its isogenic mutant strains to A549 cells. (g) The adhesion force of *S. aureus* SA113 and its isogenic mutant strains to A549 cells. (h) The murine nasal colonization abilities of *S. aureus* N315 and its isogenic mutant strains. The data shown is representative of three individual experiments performed in triplicate. One-way ANOVA was used to analyze the statistical difference between three groups after passing the normal distribution test (a, b, f, g, and h). Bars represent means and standard deviations. *p < 0.05, **p < 0.01, ***p < 0.001, ****p < 0.0001.

Previous studies revealed that *cwrA* gene was upregulated with a decrease in sensitivity to vancomycin in VISA strain [7,8]. We found that the vancomycin-intermediate *S. aureus* USA300 (LAC)-1 (with an MIC of 8 μg/ml, obtained by inducing resistance in vitro) exhibited enhanced biofilm formation and increased expression of *cwrA*, *vraR* and *vraS* genes (Supplementary Figure 3) compared to the vancomycin-sensitive strain USA300 (LAC) (with an MIC of 0.5 μg/ml). To test the hypothesis that *cwrA* is responsible for biofilm formation, we generated *cwrA* deletion mutants in *S. aureus* N315 and SA113 backgrounds and performed a 96-well format crystal violet-based biofilm assay to examine the ability of these strains to form biofilms in vitro. As shown in Figures 2a,b, Δ*cwrA* mutants reduced biofilm formation compared to the parental strains (OD_600_ decreased from 2.93 ± 0.16 to 2.22 ± 0.40 in N315, and 2.87 ± 0.03 to 2.12 ± 0.12 in SA113), and the biofilm-defective phenotype was complemented by supplying *cwrA* on the pLi50 plasmid (OD_600_ 2.84 ± 0.20 for N315-Δ*cwrA*-C, and 2.35 ± 0.19 for SA113-Δ*cwrA*-C). Subsequently, we stained the 24 h mature biofilm with SYTO9 (stains viable bacteria with green) and propidium iodide (PI, stains dead bacteria with red) reagents, and the stained biofilm was observed in more detail using CLSM. We observed a decrease in the number of live cells adhered to glass dishes in the Δ*cwrA* mutants compared to the parental strains (Figure 2c). However, there were no differences in the number of dead cells between groups. In addition, the growth curves for the WT strains, Δ*cwrA* mutants, and the *cwrA* complementary mutants grown in tryptic soy broth at 37 °C were determined, and the results showed no significant difference among these strains (Figure 2d,e). These findings illustrated that deletion of *cwrA* impaired biofilm formation in vitro.

### ΔcwrA mutants reduced the adhesion force to A549 cells

*S. aureus* is one of the major pathogens responsible for community-acquired pneumonia because of its ability to adhere to the lower respiratory tract and shift from colonization to pneumonia [26]. Therefore, the force of adhesion to lung epithelial cells is a crucial factor in the induction of pneumonia by *S. aureus*. We performed cell adhesion assays to assess the importance of CwrA in *S. aureus* adherence to A549 cells. As shown in Figure 2f,g, the Δ*cwrA* mutants exhibited a decreased adhesion force to A549 cells compared to their parental strains, while the ability adhere to A549 were restored in the *cwrA* complementary strains (2.3 × 10^6^ ±2.8 × 10^5^ CFU/ml, 1.4 × 10^6^ ±3.7 × 10^5^ CFU/ml, and 2.4 × 10^6^ ±2.1 × 10^5^ CFU/ml for N315, N315-Δ*cwrA* and N315-Δ*cwrA*-C, respectively; 3.1 × 10^6^ ±1.7 × 10^5^ CFU/ml, 1.7 × 10^6^ ±2.5 × 10^5^ CFU/ml, and 3.3 × 10^6^ ±6.6 × 10^5^ CFU/ml for SA113, SA113-Δ*cwrA* and SA113-Δ*cwrA*-C, respectively).

### ΔcwrA mutants reduced the adhesion force to murine nasal tissue

It has been reported that 30%-50% healthy adults colonized by *S. aureus*, and populations with *S. aureus* colonization have a significantly higher risk of subsequent severe infection [27]. The anterior nares serve as ecological niches for *S. aureus* [28]. Bacterial adhesion to host tissues and biomaterial surfaces is the critical initial step in biofilm formation [29]. Therefore, we employed a murine nasal colonization model to assess the adhesion force of *S. aureus* to murine nasal tissue in vivo. Mice were intranasally inoculation with 50 μl suspension containing 1.5 × 10^9^ CFU *S. aureus*, and the bacterial burden in nasal tissues was counted after 24 h. As shown in Figure 2h, the N315-Δ*cwrA* strain markedly reduced its adhesion force to murine nasal tissue compared to that of its parental strain (*p* < 0.01). This suggested that CwrA is responsible for *S. aureus* colonization in the nasal cavity of a murine model.

### Reduced biofilm formation in ΔcwrA mutant is correlated with PIA

**Figure 3.**
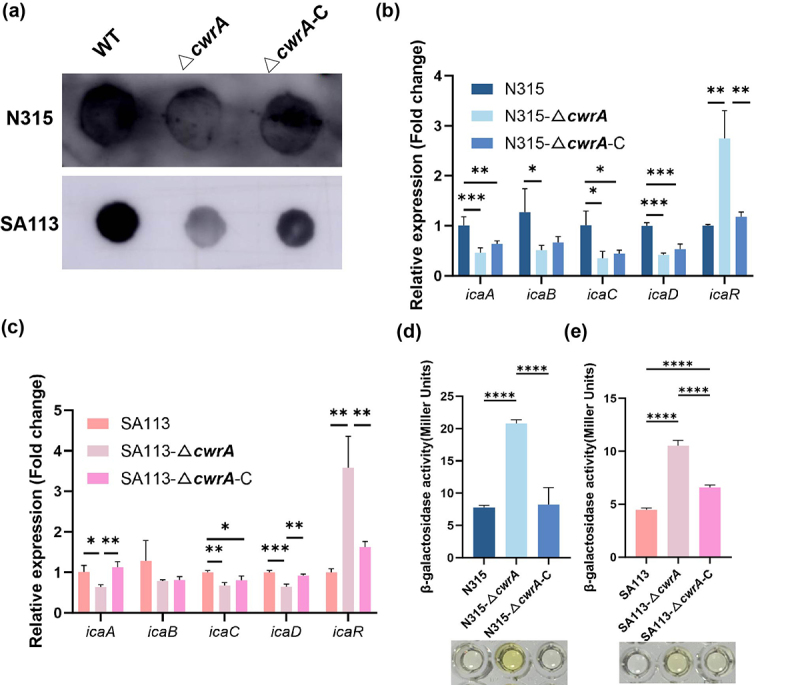
CwrA mediated biofilm formation is dependent of PIA production and regulation of *ica* locus. (a) Dot bot assay semi-quantified PIA production in tested strains. (b-c) RT-qPCR analysis for the *ica* operon transcription levels in *S. aureus* N315, SA113 and their isogenic mutant strains. Transcript levels were normalized to internal reference gene *gyrB*. (d-e) the β-galactosidase activities of *icaR* promoter in *S. aureus* N315, SA113 and their isogenic mutant strains. The data shown is representative of three individual experiments performed in triplicate. One-way ANOVA was used to analyze the statistical difference between three groups after passing the normal distribution test (b, c, d, and e). Bars represent means and standard deviations. *p < 0.05, **p < 0.01, ***p < 0.001, ****p < 0.0001.

The EPS produced by *S. aureus* contains both positively and negatively charged groups as well as hydrophobic groups. PIA is the major positively charged group that is important for intercellular adhesion and biofilm formation [17]. Since a previous study reported that VraR promotes biofilm formation in a PIA-dependent manner in *Staphylococcus epidermidis* [16], we were interested in determining whether CwrA also stimulates *S. aureus* biofilm formation in a PIA-dependent manner. Hence, we quantified the levels of extracellular PIA by immunoblot analysis. As shown in Figure 3a, PIA production in the Δ*cwrA* mutants was lower than that in the parental strains, and these changes could be partially restored by *cwrA* complementation. The *ica* (intercellular adhesion) locus is necessary for the biosynthesis of fully functional PIA and contains four different genes, *icaA*, *icaD*, *icaB* and *icaC*, which are arranged in an operon [30]. The dedicated regulator IcaR is encoded upstream of the *icaADBC* operon and it can bind to a specific DNA region upstream of *icaA* resulting in powerful suppression of *icaADBC* transcription [31]. To determine whether CwrA affects biofilm formation by regulating the expression of the *ica* operon, we determined the transcript levels of *icaA*, *icaB*, *icaC*, *icaD* and *icaR*. RT-qPCR results showed that the expression level of *icaR* was significantly higher in the Δ*cwrA* mutants than in the WT strains, while the transcript levels of *icaA*, *icaB*, *icaC* and *icaD* were downregulated in the Δ*cwrA* mutants (Figure 3b,c). Furthermore, we constructed the reporter plasmid pCL-*icaR* and performed a β-galactosidase assay to confirm the positive effect of *cwrA* on the expression of *icaR*. The β-galactosidase activities in the Δ*cwrA* mutants were significantly higher than those in the parental strains, which was consistent with the results of qRT-PCR (Figure 3d,e). Taken together, these results indicated that CwrA promotes biofilm formation by influencing the activity of the *ica* operon in *S. aureus*.

### Reduced biofilm formation in ΔcwrA mutant is correlated with eDNA

**Figure 4.**
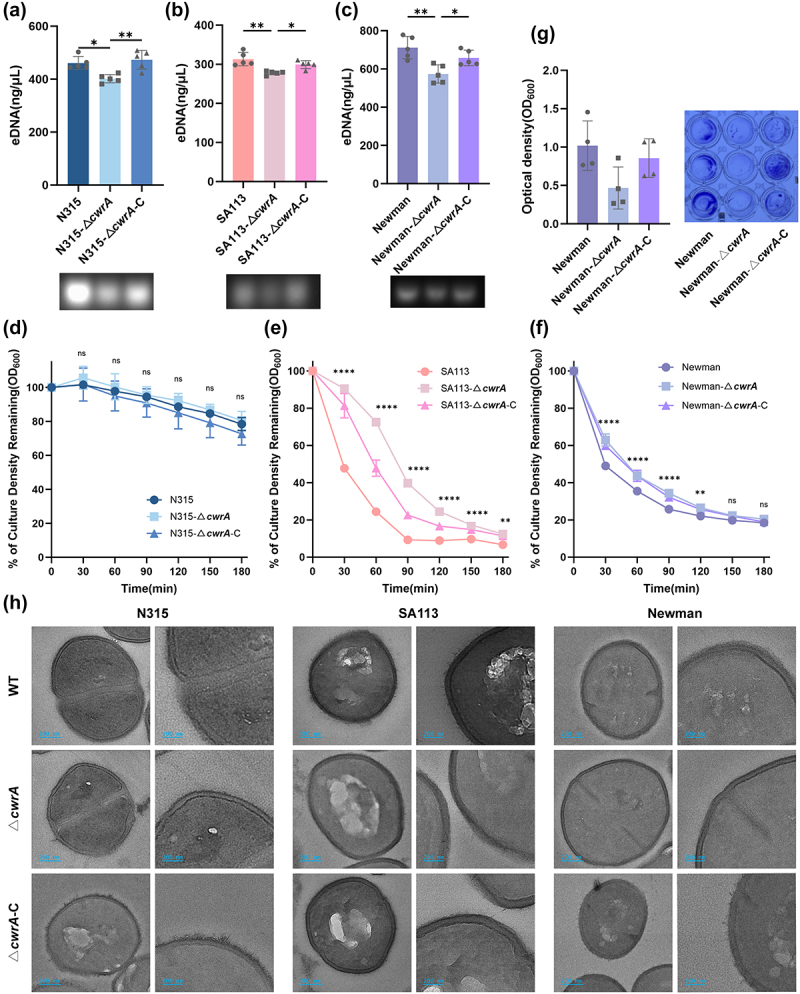
CwrA mediated biofilm formation is dependent of eDNA content in the biofilm. (a)-(c) the amount of eDNA present in the biofilms of the WT strains, the Δ*cwrA* mutant strains and the *cwrA* complementary strains. (d)-(f) autolysis of the WT strains, the Δ*cwrA* mutant strains and the *cwrA* complementary strains at 37 °C in tris-HCl buffer containing 0.05% triton X-100, and the optical density values at 600 nm were measured every 30 min. (g) The biofilm formation ability of *S. aureus* Newman and its isogenic mutant strains on polypropylene 96-well plates (right), and the optical density values at 600 nm were measured after the biofilms were dissolved with acetic acid (left). (h) Transmission electron micrograph of the WT strains, the Δ*cwrA* mutant strains and the *cwrA* complementary strains grown to mid-logarithmic phase (scale bars, 200 nm). The data shown is representative of three individual experiments performed in triplicate. One-way ANOVA was used to analyze the statistical difference between three groups after passing the normal distribution test (a, b, c, and g). Two-tailed Student’s *t* test was used to analyze the statistical difference in autolysis between the WT strains and Δ*cwrA* mutant strains at every time point (d, e, and f). Bars represent means and standard deviations. *p < 0.05, **p < 0.01, ***p < 0.001, ****p < 0.0001.

In addition to PIA, extracellular DNA (eDNA) is another important structural component of the biofilm matrix and plays a crucial role as a “glue” within the bacterial community [32]. To clarify whether variations in biofilm formation corresponded with changes in the amount of eDNA in the matrices of these biofilms, we quantified the levels of eDNA present in each biofilm. As shown in Figure 4a,b,c, the amount of eDNA present in the biofilm of the Δ*cwrA* mutants was lower than that of the WT strains, and these alterations were successfully restored in the complementary strains. Given that cell lysis is a major source of eDNA in biofilms, we assessed the autolysis ability of Triton X-100-induced autolysis assay [33]. No detectable difference in autolysis activity was observed for N315 strain (Figure 4d). However, the SA113-Δ*cwrA* mutant strain showed reduced Triton X-100-induced autolysis compared to the WT strain (Figure 4e). Previous research has indicated that MRSA strains tend to possess thicker cell walls and septa than MSSA strains [34]. Thus, we speculate that the differences in cell wall structure may explain why the alterations in autolysis activity observed in N315 (a MRSA strain with an oxacillin MIC ≥4 μg/ml) were not as pronounced as those observed in SA113 (a MSSA strain with an oxacillin MIC ≤0.25 μg/ml). To verify this hypothesis, we constructed a Δ*cwrA* mutant on another MSSA strain background. As shown in Figures 4f,c the Newman-Δ*cwrA* mutant also displayed reduced autolytic activity and a lower abundance of eDNA compared to the WT strain. We also conducted a 96-well format crystal violet-based biofilm assay to compare the biofilm formation abilities of Newman and its mutants. However, no significant difference was observed (Figure 4g), which could be attributed to the weak adhesion ability of Newman in vitro. Furthermore, transmission electron microscopy (TEM) was performed to determine whether the absence of *cwrA* affects cell morphology. In the wild-type strain samples, rough-shaped cells with unstructured components protruding from the cell surface were observed (Figure 4h). Interestingly, the cell surfaces of the Δ*cwrA* mutants were smoother, and uniformly shaped bacteria with intact cell walls were clearly visible (Figure 4h), and this phenotypic change was fully restored by supplying *cwrA* on the pLi50 plasmid. In addition, no distinction for cell wall thickness and the cytoplasmic membrane was observed. In conclusion, our findings suggest that CwrA may promote *S. aureus* biofilm formation by enhancing cell lysis and increasing the amount of eDNA present in the biofilm.

### CwrA attenuates the *S. aureus* virulence in vivo

**Figure 5.**
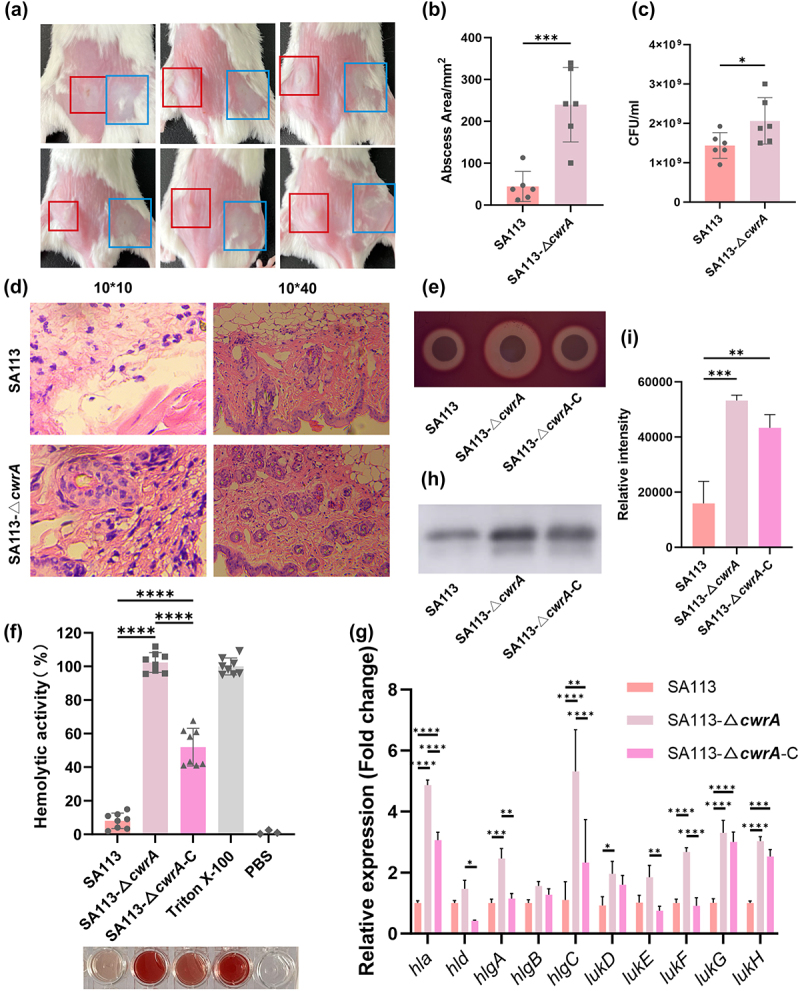
CwrA attenuates the virulence of *S. aureus* by the inhibition of hemolytic activity. (a) The mouse skin abscess after 24 h inoculation of SA113 or SA113-Δ*cwrA*. The skin abscess caused by the infection of SA113 strain were marked by red boxes, while the skin abscess caused by the infection of SA113-Δ*cwrA* strain were marked by blue boxes. (b) The abscess areas of respective strains were calculated by using the formula (A = *Π* × (*L* × *W*)) after 24 h inoculation. (c) Bacterial load in abscess homogenates of respective strains was determined by serial dilutions and spotted on TSA plates. (d) The lesion degree of skin abscess was visualized by H&E staining. (e) The hemolytic zones of *S. aureus* SA113 and its isogenic mutant strains on 5 % (vol/vol) sheep blood agar plates. (f) Erythrocyte lysis capacities of *S. aureus* SA113 and its isogenic mutant strains. PBS and 0.5 % (vol/vol) triton X-100 were used as negative and positive controls, respectively. (g) RT-qPCR analysis for the transcription levels of genes coded membrane-damaging toxins in *S. aureus* SA113 and its isogenic mutant strains. Transcript levels were normalized to internal reference gene *gyrB*. (h) Detect the secretion of α-hemolysin using the western blot assay. (i) Evaluation of grey values of α-hemolysin secretion using image J software. A two-tailed Student’s *t* test was used to analyze the statistical difference between *S. aureus* SA113 and SA113-Δ*cwrA* mutant strains (b and c). One-way ANOVA was used to analyze the statistical difference between three groups after passing the normal distribution test (a, f, g, and i). Bars represent means and standard deviations. *p < 0.05, **p < 0.01, ***p < 0.001, ****p < 0.0001.

*S. aureus* cause a wide variety of acute infections, ranging from moderately severe skin infections to fatal pneumonia and sepsis, by producing numerous exotoxins [35]. To understand the importance of CwrA in the pathogenesis of skin infections, we first compared the virulence of the WT and Δ*cwrA* mutant strains using a mouse skin abscess model. After subcutaneously inoculated with 100 μl normal saline containing 1.5 × 10^9^ CFU *S. aureus* for 24 h, the abscess areas were measured. The abscess size caused by the Δ*cwrA* mutant strain was significantly larger than that caused by the WT strain (44.48 ± 32.85 mm^2^ in the SA113 strain versus 239.69 ± 81.09 mm^2^ in the SA113-Δ*cwrA* mutant strain, *p* < 0.001) (Figure 5a,b). Subsequently, we measured the CFU counts in the soft skins infected with *S. aureus* using gradient dilution. As shown in Figure 5c, the bacterial burden in the Δ*cwrA* mutant group was significantly higher than that in the WT group (*p* < 0.05). Hematoxylin and eosin (H&E) staining showed severe inflammatory exudation and tissue necrosis in the Δ*cwrA* mutant group (Figure 5d). Overall, our results indicated that CwrA plays a critical role in skin infections caused by *S. aureus*.

### CwrA attenuates the S. aureus virulence by inhibiting the hemolytic activity

*S. aureus* secretes various toxins that not only cause tissue damage but also promote the spread and acquisition of nutrients [35]. Among these toxins, hemolysins are known for their ability to damage the membranes of a wide range of host cells and induce apoptosis [35]. To verify whether CwrA attenuated *S. aureus* virulence by inhibiting its hemolytic activity, a hemolytic activity assay was performed. We found that the SA113-Δ*cwrA* mutant strain exhibited a larger hemolytic zone on sheep blood agar plates than the WT strain after 24 h of incubation (Figure 5e). To quantitatively compare hemolytic activity, human erythrocytes were used to perform hemolytic assays, and the proportion of hemolytic activity was calculated by measuring the optical density at 600 nm. As shown in Figure 5f, the SA113-Δ*cwrA* mutant strain demonstrated a notable enhancement in its ability to lyse human erythrocytes when compared to the WT strain, and hemolytic activity levels were partially restored in the *cwrA* complementary strain (8.03 % ± 4.30 % for SA113, 102.39 % ± 5.52 % for SA113–Δ*cwrA*, 51.94% ± 10.44 % for SA113-Δ*cwrA*-C). In addition, RT-qPCR results revealed that the SA113-Δ*cwrA* mutant strain exhibited higher expression levels of critical membrane-damaging toxins, such as α-hemolysin (Hla), γ-hemolysins (HlgAB, HlgCB), δ-hemolysins (Hld), and leukotoxins (LukED, LukGH, and Panton-Valentine Leukocidin), against human erythrocytes and leukocytes (Figure 5g). Among these upregulated hemolysins, α-hemolysin is the most prominent cytotoxin which is capable of lysing red blood cells and leukocytes [36]. Therefore, we performed western blotting to further clarify whether the SA113-Δ*cwrA* mutant strain exhibited high hemolytic activity owing to high *hla* expression. As shown in Figures 5h,i, a higher level of α-hemolysin was observed in the SA113-Δ*cwrA* mutant strain than in the WT strain. Together, these results demonstrate that CwrA plays an important role in the virulence of *S. aureus*, which may be related to the suppression of hemolytic activity.

### Comparative transcriptome profiles of the ΔcwrA mutant and the wide-type

**Figure 6.**
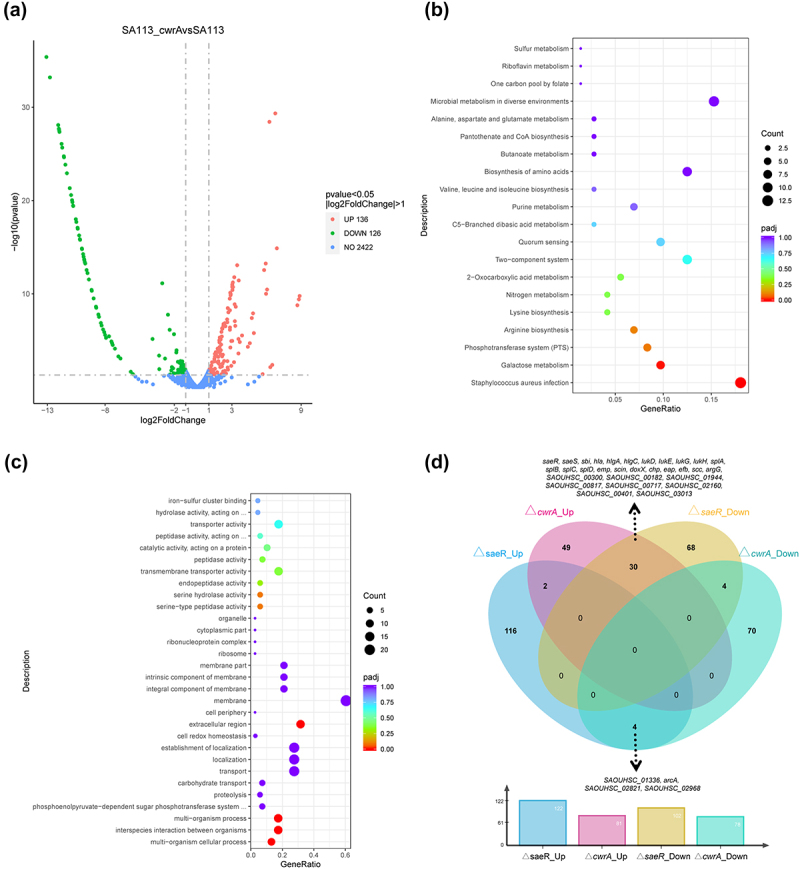
Transcriptome analysis following *cwrA* gene knockout in *S. aureus* SA113. (a) The volcano plot of transcriptome sequencing results. Each dot represents an open reading frame, with upregulated genes shown in red and downregulated genes shown in green. Adjusted P-value ≤0.05 and log2 (Fold change) ≥1 was set as the threshold for significantly differential expression. (b) Enrichment plots of the KEGG pathway analysis. (c) Enrichment plots of the GO pathway analysis. (d) A venn diagram showing the differentially expressed genes from the transcriptome analyses after the knockout of the *cwrA* and *saeR* genes. Adjusted P-value ≤0.05 and log2 (Fold change) ≥2 was set as the threshold to show the overlapping cluster of genes that exhibited significant changes in both the *cwrA* and *saeR* mutants. This venn diagram was drew by jvenn [37].

To gain further insight into the molecular mechanisms by which CwrA affects biofilm formation and virulence in *S. aureus*, the global gene expression profiles of the SA113 and SA113-Δ*cwrA* mutant strains were determined via transcriptome sequencing (RNA-Seq). A total of 262 differentially expressed genes were identified by adjusted p < 0.05 and fold change log2 > 1. Compared to the SA113 strain, 136 genes were upregulated and 126 genes were downregulated in the SA113-Δ*cwrA* mutant (Figure 6a). KEGG and GO analyses were performed to determine the biological functions of the 262 differentially expressed genes. KEGG analysis revealed that the altered genetic pathways were mainly enriched in *S. aureus* infection, galactose metabolism, phosphotransferase system, and arginine biosynthesis (Figure 6b), while in GO analysis they were mainly enriched in multi-organism cellular process, interspecies interaction between organisms, extracellular region and multi-organism process (Figure 6c). Table 1 shows a few selected categories of genes, such as those involved in regulatory processes, virulence factors, and metabolism, that were altered in the Δ*cwrA* mutant versus the parental strain.

**Table 1.** Summary of genes up-regulated by *cwrA* mutation.

Gene category	Locus	Gene name	Description	Log_2_ (Fold change)
Regulatory processes				
	SAOUHSC_01389	*pstS*	Phosphate-binding protein; PstS	6.5
	SAOUHSC_00715	*saeR*	Response regulator; SaeR	2.2
	SAOUHSC_00714	*saeS*	Sensor histidine kinase; SaeS	2.1
	SAOUHSC_00233	*lrgB*	Antiholin-like protein; LrgB	2.0
	SAOUHSC_00232	*lrgA*	Murein hydrolase regulator; LrgA	1.6
Virulence				
	SAOUHSC_01938	*splD*	Serine protease; SplD	3.6
	SAOUHSC_01115	*scc*	Fibrinogen-binding protein precursor-like protein	3.3
	SAOUHSC_01114	*efb*	Fibrinogen-binding protein; Efb	3.2
	SAOUHSC_01939	*splC*	Serine protease; SplC	3.2
	SAOUHSC_02161	*eap*	Extracellular adherence protein; Eap	3.1
	SAOUHSC_02243	*lukH*	Leukocidin-like protein; LukH	3.1
	SAOUHSC_01941	*splB*	Serine protease; SplB	3.0
	SAOUHSC_02241	*lukG*	Leukocidin-like protein; LukG	3.0
	SAOUHSC_02169	*chs*	Chemotaxis inhibitory protein	2.9
	SAOUHSC_01936	*splE*	Serine protease; SplE	2.9
	SAOUHSC_01942	*splA*	Serine protease; SplA	2.9
	SAOUHSC_01121	*hla*	α-hemolysin; Hla	2.9
	SAOUHSC_01935	*splF*	Serine protease; SplF	2.6
	SAOUHSC_02708	*hlgA*	γ-hemolysin subunit; HlgA	2.4
	SAOUHSC_00182	SAOUHSC_00182	hypothetical protein	2.3
	SAOUHSC_02167	*scin*	Staphylococcal complement inhibitor; SCIN	2.3
	SAOUHSC_02709	*hlgC*	γ-hemolysin subunit; HlgC	2.2
	SAOUHSC_01954	*lukD*	Leukocidin subunit; LukD	2.2
	SAOUHSC_01955	*lukE*	Leukocidin subunit; LukE	2.1
	SAOUHSC_00816	*emp*	Extracellular matrix protein-binding protein; Emp	2.1
	SAOUHSC_02706	*sbi*	Second immunoglobulin-binding protein; Sbi	2.1
	SAOUHSC_02710	*hlgB*	γ-hemolysin subunit; HlgB	2.0
	SAOUHSC_02260	*hld*	δ-hemolysin; Hld	1.3
	SAOUHSC_00818	*nuc*	Nuclease; Nuc	1.2
Metabolism				
Galactose metabolism				
	SAOUHSC_02452	*lacD*	Tagatose 1,6-diphosphate aldolase; LacD	3.1
	SAOUHSC_02450	*lacE*	PTS system lactose-specific transporter subunit IIBC; LacE	3.0
	SAOUHSC_02451	*lacF*	PTS system lactose-specific transporter subunit IIA; LacF	2.8
	SAOUHSC_02449	*lacG*	6-phospho-beta-galactosidase; LacG	2.8
	SAOUHSC_02453	*lacC*	Tagatose-6-phosphate kinase; LacC	2.7
	SAOUHSC_02454	*lacB*	Galactose-6-phosphate isomerase subunit; LacB	2.4
	SAOUHSC_02455	*lacA*	Galactose-6-phosphate isomerase subunit; LacA	1.9
Arginine biosynthesis				
	SAOUHSC_00899	*argG*	Argininosuccinate synthase; ArgG	6.8
	SAOUHSC_00898	*argH*	Argininosuccinate lyase; ArgH	6.3
2-Oxocarboxylic acid metabolism				
	SAOUHSC_02281		Dihydroxy-acid dehydratase	1.7
Nitrogen metabolism				
	SAOUHSC_02671	*narK*	Putative nitrate transporter; NarK	1.9
	SAOUHSC_02679	*narJ*	Probable nitrate reductase molybdenum cofactor assembly chaperone; NarJ	1.2
	SAOUHSC_02684	*nasD*	Assimilatory nitrite reductase; NasD	1.1
	SAOUHSC_02680	*narH*	Nitrate reductase beta chain; NarH	1.1

Well consistent with our finding that the Δ*cwrA* mutant exhibited decreased levels of eDNA release in biofilm, the transcription levels of a critical two-component system, *lrgAB* that influences the autolysis phenomenon in biofilm, were significantly increased in the Δ*cwrA* mutant versus the parental strain. Previous studies have reported that the *lrgAB* operon was important for inhibiting murein hydrolase activity [38]. In our study, the RNA-Seq results demonstrated that the expression of two vital autolysins encoded by *lytO* and SAOUHSC_02023 were significantly decreased (Table 2). It indicated that the Δ*cwrA* mutant decreased eDNA levels in biofilms by inhibiting murein hydrolase activity.

**Table 2.** Summary of genes down-regulated by *cwrA* mutation.

Gene category	Locus	Gene name	Description		Log_2_ (Fold change)	
Murein hydrolase						
	SAOUHSC_02019	*lytO*		Autolysin; LytO		−12.0
	SAOUHSC_02023	SAOUHSC_02023		Bifunctional autolysin		−11.7
Metabolism						
Arginine biosynthesis						
	SAOUHSC_02969	*arcA*		Arginine deiminase; ArcA		−3.0
	SAOUHSC_02968	*arcB2*		Ornithine carbamoyl-transferase; ArcB2		−2.0
	SAOUHSC_02561	*ureC*		Urease subunit alpha; UreC		−1.3
Lysine biosynthesisand 2-Oxocarboxylic acid metabolism						
	SAOUHSC_01396	SAOUHSC_01396		4-hydroxy-tetrahydrodipicolinate synthase		−2.8
	SAOUHSC_02468	SAOUHSC_02468		Acetolactate synthase		−2.5
	SAOUHSC_01394	SAOUHSC_01394		Aspartate kinase		−1.6
	SAOUHSC_01319	SAOUHSC_01319		Aspartate kinase		−1.4

Notably, the RNA-Seq profiles showed that the *saeRS* regulatory system was upregulated in the Δ*cwrA* mutant (Table 1). The SaeRS system is a key factor in the pathogenesis of *S. aureus* infections and controls the expression of approximately 40 genes, most of which encode virulence factors such as adhesins, toxins, and immune evasion proteins [39–41]. The Wayne diagram showed that 30 SaeRS-dependent genes exhibited significantly higher expression levels in the Δ*cwrA* mutant (Figure 6d). Among these 30 upregulated virulence genes in the Δ*cwrA* mutant, seven genes were related to hemolysins (*hla*, *hlgA*, *hlgC*, *lukD*, *lukE*, *lukG*, *lukH*), four genes were related to proteases (*splA*, *splB*, *splC*, *splD*), four genes were related to the cell wall (*emp*, *eap*, *efb*, *sbi*), and three genes were related to the immune response (*chp*, *scin*, *scc*) (Figure 6d). Therefore, it is reasonable to propose that CwrA attenuates the virulence of *S. aureus* by inhibiting the SaeRS system activity.

### CwrA attenuates the S. aureus virulence by inhibiting the SaeRS system

**Figure 7.**
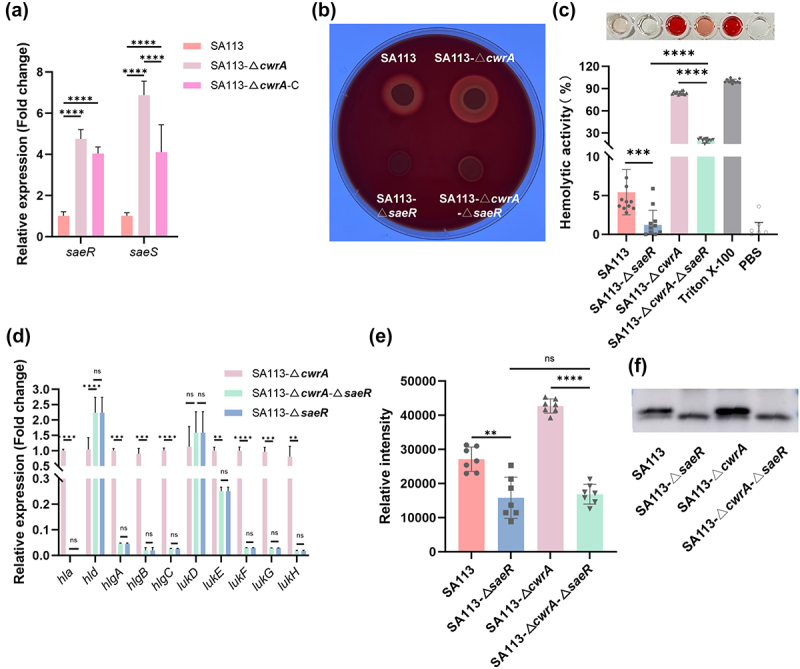
Effect of the SaeRS system on the molecular mechanism of CwrA leads to the suppression of hemolytic activity in *S. aureus*. (a) RT-qPCR analysis for the transcription levels of *saeRS* in *S. aureus* SA113 and its isogenic mutant strains. Transcript levels were normalized to internal reference gene *gyrB*. One-way ANOVA was used to analyze the statistical difference between three groups after passing the normal distribution test. (b) The hemolytic zones of *S. aureus* SA113 and its isogenic mutant strains on 5 % (vol/vol) sheep blood agar plates. (c) Erythrocyte lysis capacities of *S. aureus* SA113 and its isogenic mutant strains. PBS and 0.5 % (vol/vol) triton X-100 were used as negative and positive controls, respectively. (d) RT-qPCR analysis for the transcription levels of genes coded membrane-damaging toxins in *S. aureus* SA113-Δ*cwrA* mutant, SA113-Δ*cwrA*-Δ*saeR* mutant and SA113-Δ*saeR* mutant. Transcript levels were normalized to internal reference gene *gyrB*. (e) Evaluation of grey values of α-hemolysin secretion using image J software. (f) Detect the secretion of α-hemolysin using the western blot assay. A two-tailed Student’s *t* test was used to analyze the statistical difference between two groups (c, d, and e). Bars represent means and standard deviations. *p < 0.05, **p < 0.01, ***p < 0.001, ****p < 0.0001; ns, no significance.

Based on our RNA-seq results, we observed significant changes in the transcriptional levels of various virulence factors regulated by the SaeRS system (Table 1), a critical two-component system involved in *S. aureus* exoprotein expression [39]. In addition, RT-qPCR results further confirmed that *saeRS* was upregulated in the SA113-Δ*cwrA* strain compared to its parental strain (Figure 7a). This intriguing finding led us to hypothesize that CwrA attenuates the virulence of *S. aureus* by inhibiting the SaeRS system activity. To confirm this hypothesis, we constructed a Δ*saeR* mutant in the SA113-Δ*cwrA* genetic background and compared the hemolytic activities of SA113 and its mutants. As shown in Figure 7b, the hemolytic zone formed by SA113-Δ*cwrA*-Δ*saeR* mutant was smaller than that formed by SA113-Δ*cwrA* mutant. Unexpectedly, the hemolytic zone produced by the SA113-Δ*cwrA*-Δ*saeR* mutant was slightly larger than that produced by the SA113-Δ*saeR* mutant. What’s more, the SA113-Δ*cwrA*-Δ*saeR* mutant exhibited reduced ability to lyse human erythrocytes compared to the SA113-Δ*cwrA* mutant, although it remained stronger than SA113-Δ*saeR* mutant (5.44 % ± 2.79 % for SA113, 1.21 % ± 1.82 % for SA113-Δ*saeR*, 84.29 % ± 2.01 % for SA113-Δ*cwrA*, 19.23 % ± 2.95 % for SA113-Δ*cwrA*-Δ*saeR*) (Figure 7c). This indicated that CwrA attenuated the virulence of *S. aureus* partially because of the inhibition of the SaeRS system.

RT-qPCR was also performed to compare the expression levels of virulence factors associated with hemolytic activity. As shown in Figure 7d, the transcriptional levels of all membrane-damaging toxins except Hld in the SA113-Δ*cwrA*-Δ*saeR* mutant were comparable to those in the SA113 -Δ*saeR* mutant. In addition, western blotting results showed no distinct difference in the expression levels of Hla between the SA113-Δ*cwrA*-Δ*saeR* mutant and the SA113 -Δ*saeR* mutant (Figure 7e,f). It is reasonable to hypothesize the enhanced hemolytic activity of the SA113-Δ*cwrA*-Δ*saeR* mutant compared to the SA113 -Δ*saeR* mutant was due to the increased δ-hemolysin production. Taken together, these results suggest that CwrA could attenuate *S. aureus* virulence partially through inhibition of SaeRS system.

## Discussion

**Figure 8.**
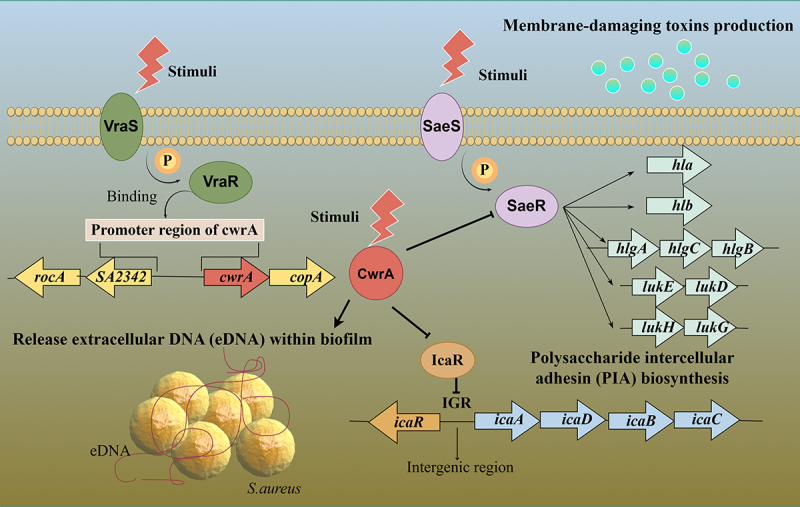
Summary diagram of the mechanisms by which CwrA influence the pathogenicity of *S. aureus*. This figure was draw by Figdraw (ID: SYPSAa6b1b).

*S. aureus* VraR protein belongs to the NarL/FixJ family and has a typical helix-turn-helix (HTH) structural motif at the C-terminal DNA-binding domain [42]. With the ability to bind to the promoter region, phosphorylated VraR can increase the expression of genes that respond to cell wall stress and play crucial roles in peptidoglycan biosynthesis and antibiotic resistance [43–46]. In this study, we demonstrated that VraR can directly bind to the promoter of a gene called *cwrA*, which is important for *S. aureus* biofilm formation and its ability to damage host erythrocytes (Figure 8).

In *S. aureus*, it has been reported that the *vraSR* deletion mutant strain (Δ*vraSR*) exhibited reduced adhesion to HeLa cells and decreased ability to survive within polymorphonuclear leukocytes (PMNs) because of its reduced biofilm-forming ability [47]. Considering the regulatory relationship between VraR and CwrA, as well as the finding that the vancomycin-resistant strain USA300 (LAC)-1 showed increased expression of *vraR*, *vraS* and *cwrA*, along with enhanced biofilm formation ability compared to its vancomycin-sensitive parental strain USA300 (LAC), we further investigated the role of CwrA in *S. aureus* biofilm formation. In this study, we found that the *cwrA* deletion mutant was defective in forming biofilms in vitro and adhering to both A549 cells and murine nasal tissue in vivo. Interestingly, these findings were consistent across both the MRSA and MSSA strains. A previous study also revealed that the deletion of *vraSR* could result in impaired biofilm formation in *S. epidermidis* by decreasing PIA production and upregulating *icaR* [16]. Here, we showed that deletion of *cwrA* caused IcaR activation and thus a reduction in the biosynthesis of PIA. IcaR has been identified as a repressor of *icaADBC* transcription in *S. aureus*. Various regulators have been reported to indirectly affect *icaADBC* transcription by affecting *icaR* expression [48–50]. Cerca et al. reported that SarA and σ^B^ were positive regulators of *icaR* expression in *S. aureus* [48]. Besides, an enhancement in PIA-dependent biofilm formation has been revealed in *spx* mutant 8325–4 strain of *S. aureus* because Spx modulates the expression of *icaR* [49]. The results reported by Cue et al. revealed that the positively regulated expression of *icaADBC* by Rbf is indirect through the inhibition of *icaR* [50]. Similarly, with a profound negative effect on the transcriptional expression and promoter activity of *icaR*, CwrA appears to be a vital protein that indirectly enhance *icaADBC* transcription by decreasing *icaR* expression.

Previous studies reported that Δ*vraSR* mutant strains exhibited a thinner cell wall than parental strains in both *S. aureus* and *S. epidermidis* [16,47]. There was no difference in the cell wall thickness between the Δ*cwrA* mutant strains and their corresponding parental strains. However, the TEM results showed that the cell surface of the Δ*cwrA* mutants was smoother than those of the WT strains, and the unstructured components on the cell surface of the WT strains disappeared in the Δ*cwrA* mutant strains. Furthermore, MSSA strains with the *cwrA* deletion exhibited weaker autolytic activity than their parental strains. The results of RNA-Seq analysis showed a significant decrease in the expression of two uncharacterized autolysins encoded by *lytO* and *SAOUHSC_02023*. Plotka et al. reported an increase in cell wall roughness in *S. aureus* ATCC25923 cells treated with a peptidoglycan hydrolytic enzyme with lytic activity [51]. Expression and preliminary activity analyses indicated that LytO has lytic activity towards live *S. aureus* [52]. *SAOUHSC_02023* encodes a bifunctional autolysin, which is involved in *S. aureus* cell division, daughter cell separation, autolysis, and stress response [53–55]. Thus, it is possible that the morphological alterations in the Δ*cwrA* mutant strains were due to the decreased lytic activities of LytO and SAOUHSC_02023, and then decreased eDNA release within the biofilm. Furthermore, RNA-Seq analysis revealed that the transcriptional level of *lrgAB* was upregulated in the Δ*cwrA* mutant strains. The *lrgAB* operon can function as an inhibitor of murein hydrolase activity [38]. Therefore, whether CwrA influences the expression levels of *lytO* and *SAOUHSC_02023* directly or indirectly by upregulating the *lrgAB* operon requires further investigation in subsequent studies.

Bacterial cells can sense changes in environmental conditions and respond accordingly. Two-component systems (TCSs) are primary machinery which contain a histidine kinase (HK) responsible for sensing extracellular stimuli and a response regulator (RR) that accepts phosphoryl signals from HK and affects changes in cellular physiology by regulating gene expression [56]. Crosstalk among different TCSs is common in *S. aureus*, and it is beneficial for *S. aureus* to adapt to environmental stresses, such as antibiotics, osmotic pressure, pH, and redox state [57]. Villanueva et al. reported that because of the sequence identity between GraS and ArlS, ArlR could be cross-phosphorylated by the HK of GraSR induced by colistin and ultimately express the ArlSR target gene *spa* [58]. Another example of crosstalk in *S. aureus* is the regulatory potential of Stk1/Stp1-mediated crosstalk between GraSR, WalKR, and VraSR TCSs, which contribute to cell wall metabolism, virulence, and antibiotic resistance [59–61]. Given the results presented here, CwrA seems to be a mediator involved in the crosstalk between VraSR and SaeRS TCSs, and contributes to attenuating the virulence of *S. aureus* by inhibiting its capability to cause host erythrocyte damage. We reported the enhancement of SaeRS transcription in the Δ*cwrA* mutant strain, but the exact role of CwrA involvement in VraSR and SaeRS and its contribution to virulence requires further investigation. Notably, the SA113-Δ*cwrA*-Δ*saeR* mutant strain exhibited slightly stronger hemolytic activity than the SA113-Δ*saeR* mutant strain. Besides, the results of RT-qPCR indicated that the transcriptional level of *hld* that codes δ-hemolysin in the SA113-Δ*cwrA*-Δ*saeR* mutant strain was higher than that in the SA113-Δ*cwrA* mutant strain. The *hld* gene is embedded within the region encoding RNAIII [62]. RNAIII is a non-coding RNA which is positively regulated by *agr* system [63] and controls the expression of virulence factors at both transcriptional and translational levels [64]. Therefore, apart from SaeRS, the essential global regulator of virulence in *S. aureus*, *agr* system may be also involved in this sophisticated regulatory network.

In conclusion, we revealed that CwrA, a CWSS regulated by VraR, plays an important role in *S. aureus* biofilm formation in a PIA and eDNA associated manner (Figure 8). We have also demonstrated that the mechanism by which CwrA influences hemolytic activity may be by regulating the transcription levels of the SaeRS system (Figure 8). As biofilm formation and hemolytic ability appear to be important in *S. aureus* infections, more in-depth investigations to elucidate the molecular pathways involved in these pathogenic processes would be helpful in developing novel anti-infection strategies. Here, we report that CwrA is a promising target for the development of drugs to treat *S. aureus* infections.

## Supplementary Material

Supplemental Material

Supplemental Material

Fig S2.tif

## Data Availability

The data that support the findings of this study are openly available in Science Data Bank (ScienceDB (scidb.cn). DOI:10.57760/sciencedb.10691.) RNA-Seq data were deposited in the NCBI Sequence Read Archive (SRA) with accession number PRJNA1113535 (Staphylococcus aureus strain:SA113 sequencing | is… (ID 1,113,535) – BioProject – NCBI (nih.gov)). *S. aureus* N315 and Newman genomes can be found at the National Center for Biotechnology Information (NCBI) with accession numbers ASM964v1 and ASM3748308v1, respectively. The reference sequence of *S. aureus* SA113 can be found at NCBI with accession numbers SAMN15745744 and SAMN18385255, and the reference sequence of *S. aureus* USA300 (LAC) can be found at NCBI with accession numbers SAMN39529698.
