# Supplementary figures and images for: The roles of cell wall inhibition responsive protein CwrA in the pathogenicity of Staphylococcus aureus
Source: Virulence. 2024 Oct 2;15(1):2411540. doi: 10.1080/21505594.2024.2411540 (PMC11457683; doi:10.1080/21505594.2024.2411540)

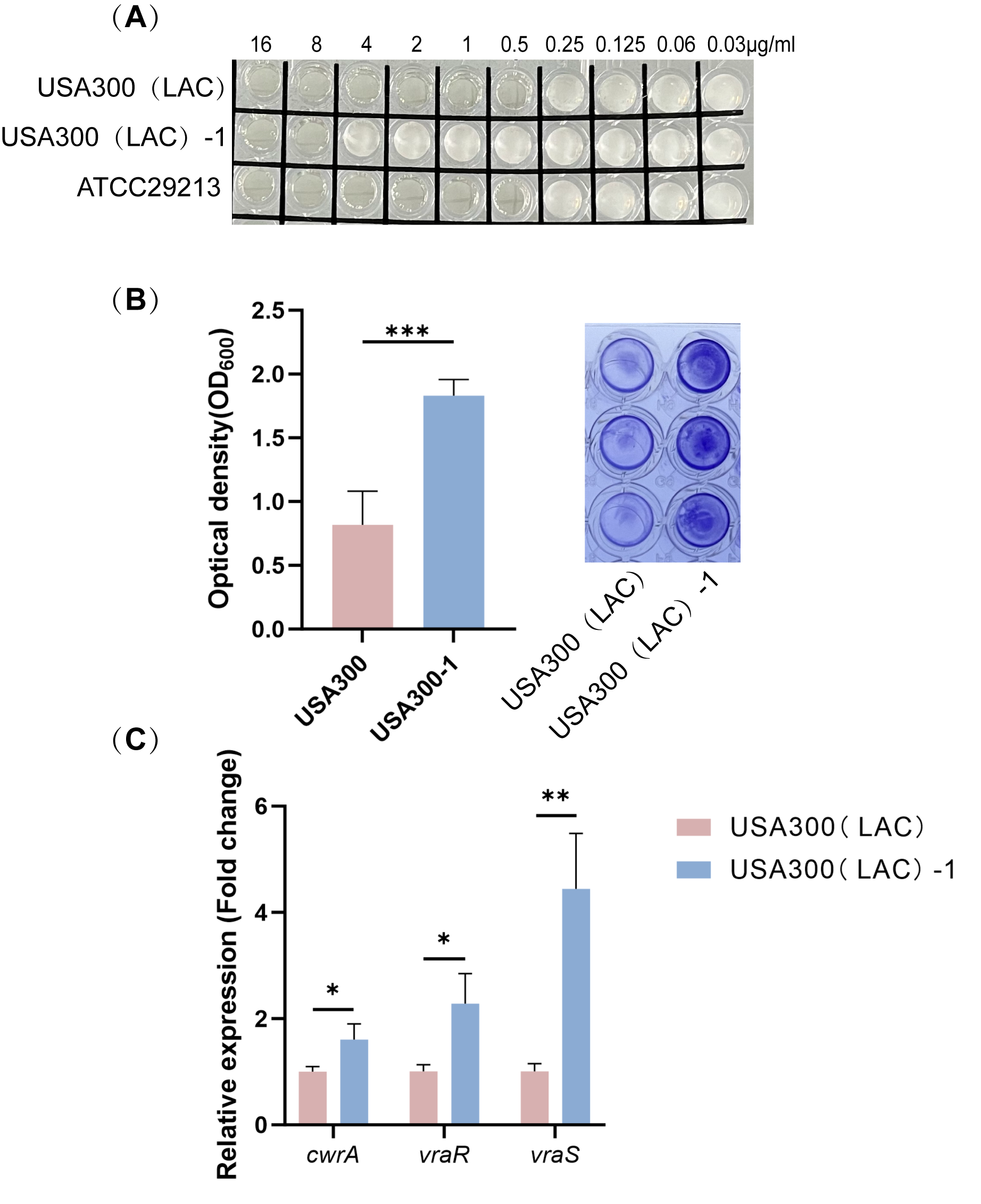

Supplement: Supplemental Material [file KVIR_A_2411540_SM3458.tif]

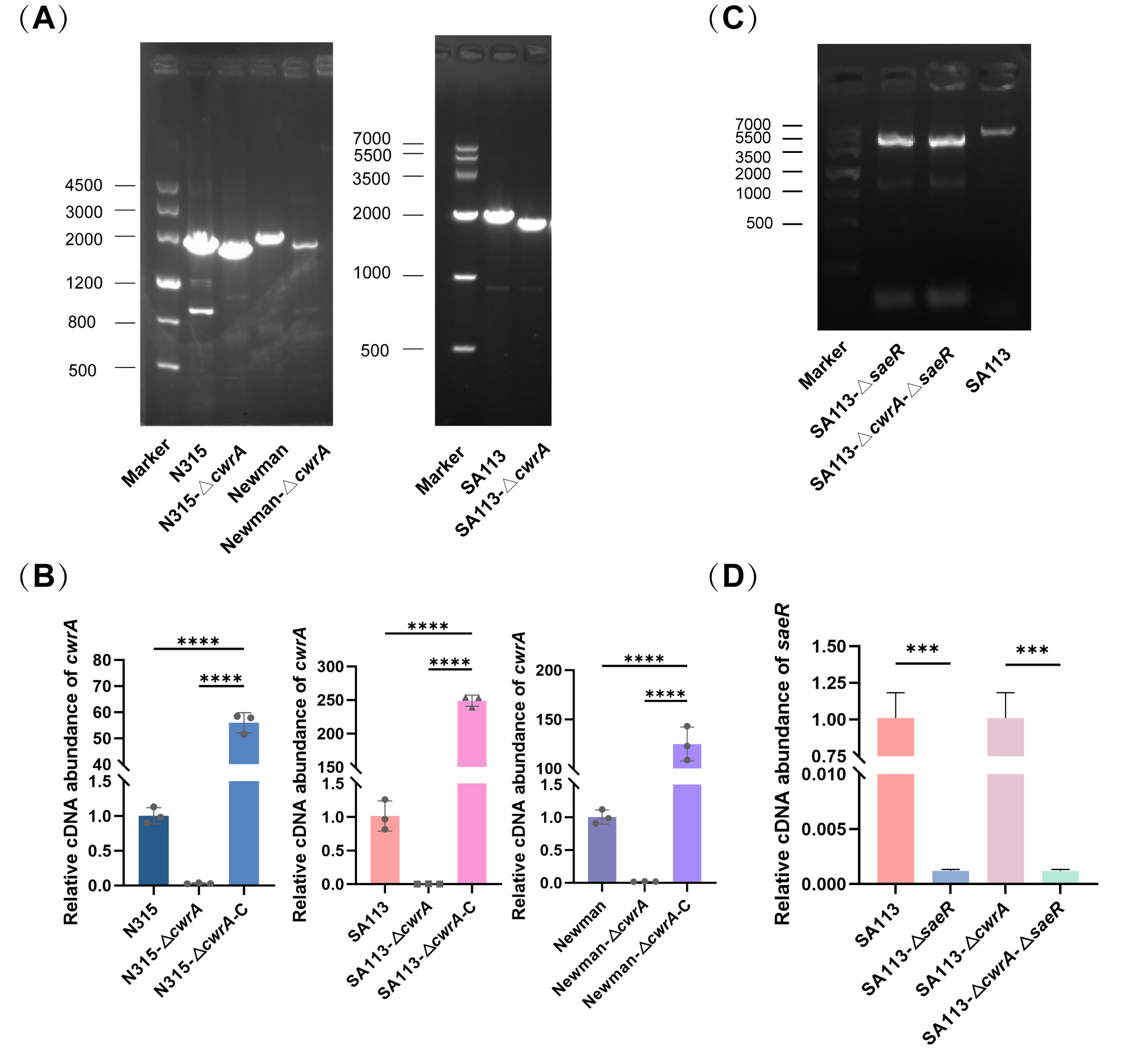

Supplement: Supplemental Material [file KVIR_A_2411540_SM3444.tif]

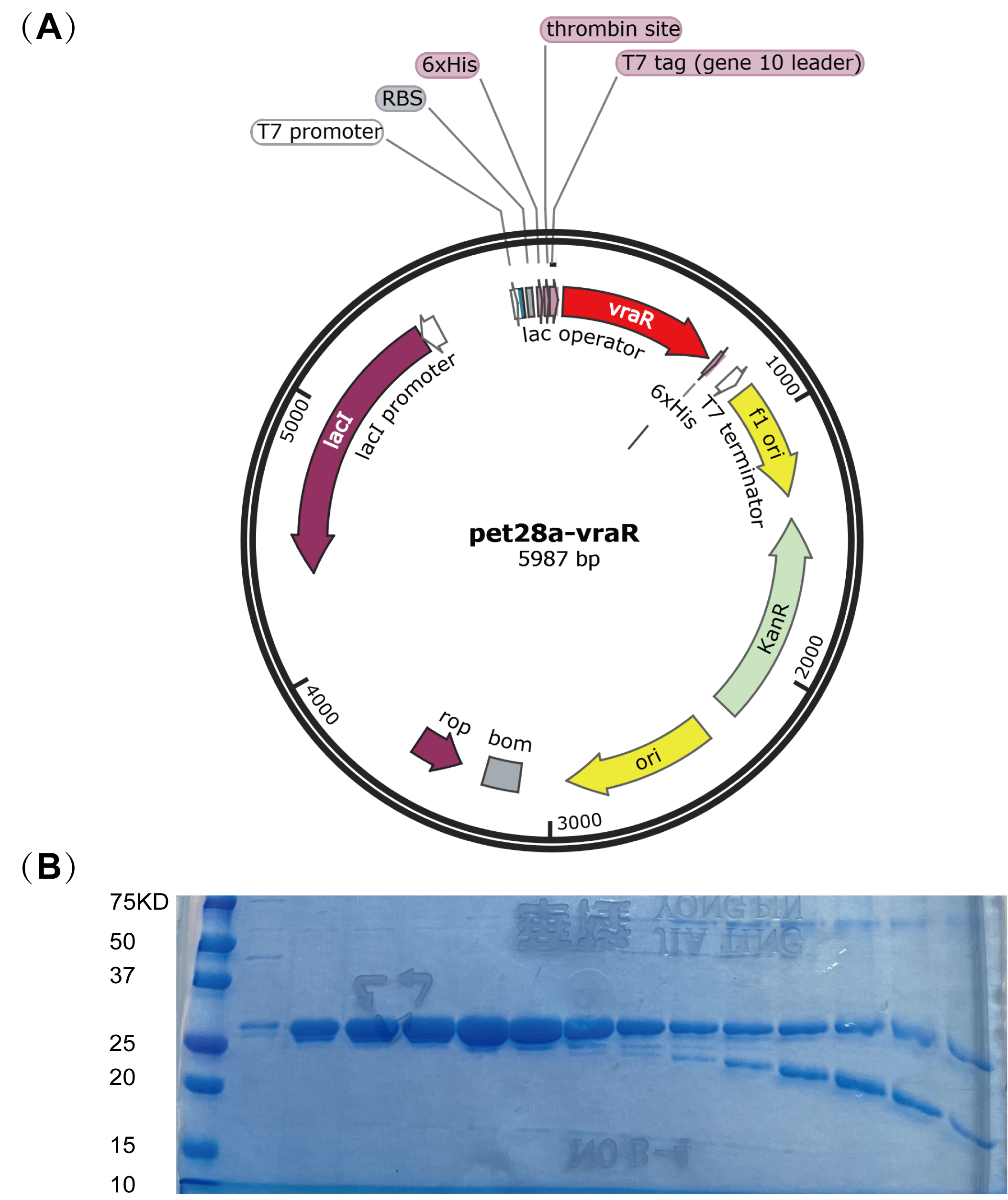

Supplement: Fig S2.tif [file KVIR_A_2411540_SM0262.tif]
